# Co-detection of members of the urokinase plasminogen activator system in tumour tissue and serum correlates with a poor prognosis for soft-tissue sarcoma patients

**DOI:** 10.1038/sj.bjc.6605520

**Published:** 2010-01-05

**Authors:** H Taubert, P Würl, T Greither, M Kappler, M Bache, C Lautenschläger, S Füssel, A Meye, A W Eckert, H-J Holzhausen, V Magdolen, M Kotzsch

**Affiliations:** 1 Department of Oral and Maxillofacial Plastic Surgery, Martin-Luther-University Halle-Wittenberg, Halle, Germany; 2 Clinic of General Surgery, University of Ulm, Ulm, Germany; 3 Department of Radiotherapy, Martin-Luther-University Halle-Wittenberg, Halle, Germany; 4 Institute of Medical Biometry and Informatics, University Halle-Wittenberg, Halle, Germany; 5 Department of Urology, Dresden University of Technology, Dresden, Germany; 6 Medizinisches Labor Ostsachsen, Dresden/Bautzen, Germany; 7 Institute of Pathology, Martin-Luther-University Halle-Wittenberg, Halle, Germany; 8 Department of Obstetrics and Gynecology, Technical University of Munich, Munich, Germany; 9 Institute of Pathology, Dresden University of Technology, Dresden, Germany

**Keywords:** urokinase plasminogen activator system, sarcoma patients, prognosis

## Abstract

**Background:**

The urokinase plasminogen activator (uPA) system is one of the best-investigated protease systems, both under physiological and pathological conditions, including various types of cancer. However, effects of co-expression of members of the uPA system in soft-tissue sarcoma (STS) patients at the protein level in both tumour tissue and serum have not been investigated yet.

**Methods:**

We examined 82 STS patients for protein levels of uPA, PAI-1and uPAR in tumour tissue and serum by ELISA.

**Results:**

A significant correlation between high antigen levels of uPA, PAI-1 or uPAR in tumour tissue, and of uPAR in serum, with poor outcome of STS patients was found for the first time. Most strikingly, we observed an additive effect of combined uPA, PAI-1 or uPAR levels in tumour tissue extracts with uPAR levels in serum on patients’ prognosis. High uPA/uPAR, PAI-1/uPAR and uPAR/uPAR antigen levels in tumour tissue/serum were associated with a 5.9-fold, 5.8-fold and 6.2-fold increased risk of tumour-related death (*P*=0.003, 0.001 and 0.002, respectively) compared with those patients who displayed low levels of the respective marker combination.

**Conclusion:**

As expression of members of the uPA system in tumour tissue and serum is additively correlated with prognosis of STS patients, our results suggest that combinations of these biomarkers can identify STS patients with a higher risk of tumour-related death.

The urokinase plasminogen activator (uPA) system comprises the serine protease uPA, its receptor uPAR and two inhibitors PAI-1 and PAI-2 (Duffy, 2004). Components of the uPA system have an important role in tumourigenesis, extracellular matrix (ECM) degradation, angiogenesis, as well as in proliferation, migration and adhesion of tumour cells (Duffy and Duggan, 2004; Mondino and Blasi, 2004; Pillay *et al*, 2007). They are prognostic factors in different types of cancer. For example, elevated tumour tissue levels of uPA have prognostic impact in a variety of cancers such as breast, colon, oesophagus, ovary and stomach cancer, high antigen levels of uPAR are associated with poor prognosis in cancer of the breast and colon, and elevated levels of PAI-1 are correlated with shortened overall and/or disease-free survival in renal, ovarian and breast cancer (Duffy and Duggan, 2004; Clark *et al*, 2008). Nevertheless, the clinical finding that an enzyme inhibitor does not have a protective function but is an indicator of worse prognosis is, at first glance, surprising. However, apart from being a uPA inhibitor, it has been demonstrated that PAI-1 has different, additional tumour-supporting functions (Duffy *et al*, 2008). Only one study has so far determined the protein expression of components of the uPA system and evaluated its impact on prognosis for soft-tissue sarcoma (STS) patients. Increasing uPA protein levels in tumour tissue were associated with local recurrence and metastasis in 69 STS patients (Choong *et al*, 1996). Up to now, however, there are no studies that have investigated protein levels of all three components of the uPA system in tumour tissue and serum of STS patients. Therefore, we determined the expression of uPA, uPAR and PAI-1 on protein level in a cohort of 82 adult STS patients, and evaluated their relationship with relevant clinicopathological parameters and overall survival (OS). In addition, the effect of combined uPA, uPAR and PAI-1 values in tumour tissue and serum of STS patients was analysed.

## Materials and methods

### Patients and tumour material

This study was performed on tumour tissue samples of 82 adult patients with histologically verified STS that have been described in previous studies (Würl *et al*, 2002). The study adhered to national regulations on ethical issues and was approved by the local ethical committee. All patients gave written informed consent (Department of Surgery 1, University of Leipzig, Germany). The median age of patients at surgery was 55.8 years (range 17–83 years). The median follow-up time of patients was 46 months (range 2–146 months after primary tumour resection). Tumours were staged according to the UICC system. Relevant data on clinical parameters of the STS patients are shown in Table 1.

Out of 82 STS patients, uPA, uPAR and PAI-1 antigen levels could be determined in tumour tissue samples for 80 patients, and for 79 patients in preoperative serum samples. For 77 patients, uPA, uPAR and PAI-1 protein levels were determined in both tumour tissue and serum.

### Determination of uPA, uPAR and PAI-1 antigen by ELISA

Tissue extracts were prepared from frozen STS in the presence of Triton X-100 (detergent extracts) as previously described (Jänicke *et al*, 1994; Luther *et al*, 1996). Briefly, after solubilisation of membrane-bound proteins using Tris buffer containing the non-ionic detergent Triton X-100 (1%), cell debris was separated by centrifugation and the supernatant was stored at −20°C until use. For each STS patient, serum samples were obtained 2 days before surgery and were stored at −80°C until assayed. The uPA, uPAR and PAI-1 antigen content levels in tissue extracts, as well as in serum of STS patients, were determined applying commercially available ELISA kits (IMUBIND uPA ELISA # 894, IMUBIND uPAR ELISA #893 and IMUBIND PAI-1 ELISA 821; American Diagnostica Inc., Stamford, CT, USA) according to the manufacturer's instructions. Antigen concentrations in tissue extracts were expressed in ng analyte per mg of total protein.

### Statistical analysis

The levels of significance between continuous variables of biological markers were calculated using Spearman's rank correlation (*r*_s_). The relationship of biological marker expression levels with clinicopathological parameters was evaluated using non-parametric Mann–Whitney or Kruskal–Wallis tests. For survival analyses, the OS of STS patients was used as follow-up end point. The association between biological marker levels and prognosis was evaluated using univariate Kaplan–Meier analyses, and the log-rank test was applied to test for differences. For multivariate analyses, Cox's proportional hazard regression model was used to calculate the relative risk and its 95% confidence interval (CI) in the analysis of OS. Multivariate models were adjusted for known clinical prognostic factors in STS patients: tumour stage, tumour type, type of tumour resection and tumour localisation. All calculations were performed using the SPSS 17.0 program (SPSS-Science, Chicago, IL, USA). All *P*-values were two-sided and *P*<0.05 was considered statistically significant.

## Results

### Correlation of uPA, uPAR and PAI-1 levels in tumour tissue and in serum of STS patients

We investigated the correlation between antigen levels of uPA, uPAR and PAI-1 in tumour tissue extracts (uPA-T, uPAR-T and PAI-1-T, respectively) and in serum (uPA-S, uPAR-S and PAI-1-S, respectively). In tumour tissue extracts, we detected a strong correlation between uPA-T and uPAR-T (*r*_s_=0.84, *P*<0.001) or PAI-1-T (*r*_s_=0.69, *P*<0.001), and between uPAR-T and PAI-1-T (*r*_s_=0.83, *P*<0.001) antigen levels (Table 2). In serum, a moderate correlation was observed between uPA-S and uPAR-S (*r*_s_=0.54, *P*<0.001). However, PAI-1-S values were only weakly, if at all, correlated with uPA-S (*r*_s_=0.24, *P*<0.05) or uPAR-S (*r*_s_=0.19, n.s.) values. Next, we evaluated the relationship between uPA, uPAR and PAI-1 antigen levels in tumour tissue extracts with that in serum (Table 2). We found a moderate, significant correlation between uPAR-S concentration and uPA-T, uPAR-T and PAI-1-T levels (*r*_s_=0.49, 0.54 and 0.46, respectively, all *P*<0.001). Furthermore, the uPA-S concentration was weakly but significantly correlated with uPA-T, uPAR-T and PAI-1-T values (*r*_s_=0.36, 0.38 and 0.31, respectively, all *P*<0.01). These results suggest that uPA, uPAR and PAI-1 levels in the tumour may affect each other or are regulated in a concerted manner and that they are strikingly related to the serum levels of uPAR protein in STS patients.

### uPA, uPAR and PAI-1 antigen levels in STS tissues and association with clinical parameters and prognosis

The amount of uPA-T, uPAR-T and PAI-1-T protein has been determined in 80 STS tissue samples and was related to the whole protein content in each sample. The median protein expression for uPA-T was 1.78 ng mg^−1^ (range: 0–22.76), for uPAR-T 3.98 ng mg^−1^ (range: 0.17–103.47) and for PAI-1-T, it was 21.43 ng mg^−1^ (range: 0.57–1,279.0). For statistical analysis, the median values were used as cutoff points to separate STS patients into groups with low or high antigen levels in tumour tissue extracts. The association of uPA-T, uPAR-T and PAI-1-T levels with relevant clinicopathological factors is summarised in Table 1. High levels of uPA-T, uPAR-T and PAI-1-T antigen were significantly associated with histological subtype (*P*<0.001, *P*<0.001 and *P*=0.006, respectively), with tumour grade (*P*<0.001, *P*<0.001 and *P*=0.001, respectively), and with tumour stage (*P*<0.001, *P*<0.001 and *P*=0.006, respectively). Moreover, uPA-T, uPAR-T and PAI-1-T antigen levels were significantly higher in patients who died during follow-up time (Table 1).

For survival analysis, the Kaplan–Meier test was performed to study the effect of uPA-T, uPAR-T and PAI-1-T antigen levels on prognosis. Overall survival was significantly different between patients’ groups with high or low antigen levels for all three markers. Patients with high *vs* low expression of uPA-T survived on an average for 44 months *vs* 86 months (*P*=0.003), patients with high *vs* low expression of uPAR-T survived on an average for 54 *vs* 76 months (*P*=0.033) and those with high *vs* low expression of PAI-1-T survived on an average for 53 *vs* 79 months (*P*=0.004) (Table 3). The independent relationship of uPA-T, uPAR-T and PAI-1-T was studied using multivariate Cox's regression analysis. For patients whose tumours expressed either a high uPA-T or a high PAI-1-T antigen level, we detected a significantly, nearly three-fold increased risk of tumour-related death (RR=2.9, 95% CI=1.1–7.7, *P*=0.032; and RR=2.6, 95% CI=1.1–6.0, *P*=0.029, respectively) compared with those patients who displayed low uPA-T or PAI-1-T values in their tumours, respectively (Table 3). On the other hand, uPAR-T levels did not significantly contribute to the base model for OS (Table 3). Therefore, only high uPA-T or PAI-1-T antigen tumour tissue levels are independent prognostic factors for OS of STS patients.

### uPA, uPAR and PAI-1 antigen concentration in serum of STS patients and association with clinical parameters and prognosis

The concentration of uPA (uPA-S), uPAR (uPAR-S) and PAI-1 (PAI-1-S) antigen in pre-operative serum samples of 79 STS patients has been measured by ELISA. We observed a median antigen concentration of 0.66 ng ml^−1^ (range: 0–4.76) for uPA-S, of 1.60 ng ml^−1^ (range: 0.24–8.03) for uPAR-S and of 1084.0 ng ml^−1^ (range: 44.0–6,068.5) for PAI-1-S. For statistical analysis, the median values uPA-S, uPAR-S and PAI-1-S were used as cutoff points to separate STS patients in groups with low or high antigen concentrations in serum. High concentrations of uPAR-S antigen were significantly associated with histological subtype (*P*=0.004), tumour grade (*P*<0.001) and tumour stage (*P*=0.019) (Table 1). On the contrary, uPA-S and PAI-1-S serum antigen levels did not show any association with clinicopathological features (Table 1).

In Kaplan–Meier analysis, a significantly different OS has been found between groups of STS patients with high or low uPAR-S concentrations. Patients with high *vs* low levels of uPAR-S survived on an average for 45 months *vs* 86 months (*P*=0.005) (Table 3). Serum levels of uPA and PAI-1 antigen were not associated with prognosis of STS patients in univariate analysis (Table 3). In multivariate Cox's regression analysis, we found that STS patients with elevated uPAR-S antigen levels possessed a 3.5-fold increased risk of tumour-related death (RR=3.5, 95% CI=1.5–8.3, *P*=0.004) compared with patients with low uPAR-S concentrations (Table 3).

### Combined analysis of uPA, uPAR and PAI-1 antigen levels for OS

We assessed whether a combination of biological markers might add prognostic information for patients’ survival. For this analysis, the patient cohort was divided into four groups on the basis of the combination of high and low marker values. In Kaplan–Meier analysis, a co-detection of high levels of uPA-T and PAI-1-T or uPAR-T, and of PAI-1-T and uPAR-T, was significantly associated with a shorter OS compared with patients with low antigen levels of the combined markers in tumour tissue (Table 4). Patients with high *vs* low values of uPA-T/uPAR-T survived on an average for 41 *vs* 75 months (*P*=0.007), those with high *vs* low expression of uPA-T/PAI-1-T survived on an average for 46 *vs* 88 months (*P*=0.004), and those with high *vs* low expression of PAI-1-T/uPAR-T survived on an average for 53 *vs* 86 months (*P*=0.014) (Table 4). In multivariate Cox's regression analysis, the subgroup of patients with high uPA-T/uPAR-T, high uPA-T/PAI-1-T or high uPAR-T/PAI-1-T antigen levels showed the worst OS with RR values of 3.3 (95% CI=1.2–9.6, *P*=0.026) 3.6 (95% CI=1.2–10.4, *P*=0.019) or 3.2 (95% CI=1.1–9.4, *P*=0.034), respectively, compared with patients having tumours with low values for both markers (Table 4).

Furthermore, the effect of uPA, PAI-1 or uPAR antigen levels in tumour tissue, in combination with the uPAR antigen concentration in serum on OS, was studied. In univariate Kaplan–Meier analysis, OS was significantly different between patient subgroups, with high values of combined markers *vs* low values for all three marker combinations, i.e., patients with high *vs* low levels of uPA-T/uPAR-S survived on an average for 44 months *vs* 94 months (*P*=0.012), those with high *vs* low levels of uPAR-T/uPAR-S on an average for 38 *vs* 80 months (*P*=0.008) and those patients with high *vs* low levels of PAI-1-T/uPAR-S on an average for 39 *vs* 82 months (*P*=0.007) (Table 4).

**Figure 1:**
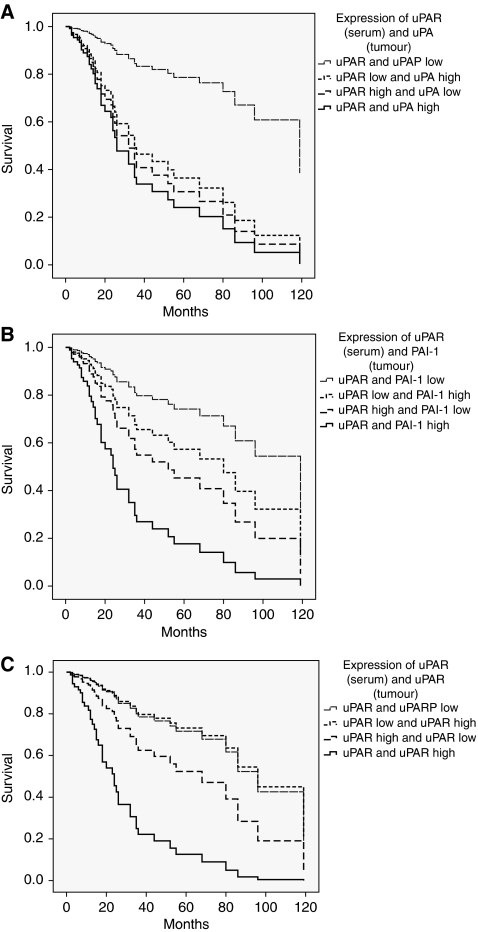
Multivariate Cox's regression hazard analysis: association of combined marker levels with overall survival of STS patients. (**A**) Combined values of uPAR antigen in serum and uPA antigen in tumour tissue extracts. (**B**) Combined values of uPAR antigen in serum and PAI-1 antigen in tumour tissue extracts. (**C**) Combined values of uPAR antigen in serum and in tumour tissue extracts. Patients with high expression of uPAR antigen levels in serum and with high levels of uPA (**A**), PAI-1 (**B**) or uPAR (**C**) in tumour tissue extracts possessed a 5.9-, 5.8- or 6.2-fold increased risk of tumour-related death compared with patients who showed a low concentration for both proteins.

In multivariate Cox's regression analysis, we found for the subgroups of patients with a high uPA-T/uPAR-S, a high uPAR-T/uPAR-S or a high PAI-1-T/uPAR-S, an ∼6-fold significantly increased risk of tumour-related death (RR=5.9, 95% CI=1.8–19.2, *P*=0.003; RR=6.2, 95% CI=1.9–20.0, *P*=0.002; RR=5.8, 95% CI=1.9–17.0, *P*=0.001, respectively) compared with those patients who showed low values for each marker combination (Table 4, Figure 1). Thus, there is an additional effect on prognosis when uPA, PAI-1 and uPAR levels in tumour tissue were combined, which is even more pronounced for the combination of uPA, PAI-1 and uPAR tissue levels with uPAR serum values.

## Discussion

The uPA system is one of the best-investigated protease systems under both physiological and pathological conditions, including various types of cancer (Duffy *et al*, 2008).

There are many studies investigating the clinical impact of expression of members of the uPA system and its correlation to prognosis in carcinoma (Duffy and Duggan 2004; McMahon and Kwaan, 2007–08; Duffy *et al.* 2008) but so far only one study has been conducted for STS patients (Choong *et al*, 1996). Choong *et al* (1996) detected an association of increasing uPA protein levels in tumour tissue with local recurrence and metastasis in 69 STS patients. In our study, single protein levels of uPA and PAI-1, and combined protein levels of uPA, uPAR and PAI-1, in tumour tissues were significantly correlated with an up to 3.6-fold increased risk of tumour-related death. There are two main reasons for a clinical impact of expression of uPA, uPAR and PAI-1 in tumour tissues and their correlation with prognosis. First, the uPA system has a role in modulating cell adhesion, overcoming ECM boundaries and can interact with potential oncogenes. The binding of uPA to membrane-bound uPAR results in focusing active uPA to cells, and in efficient cell-associated cleavage of plasminogen to plasmin, which subsequently breaks down ECM and facilitates cancer invasion (Clark *et al*, 2008). On one hand, uPAR concentrates uPA enzymatic activity to the tumour cell, on the other, there is also a mutual cooperation between uPAR and further interactors: the activity of integrins, chemokines, cytokines and growth factor receptors (Ragno, 2006; Tang and Wei, 2008). Furthermore, in cancer cells, Ras signalling is linked to the uPA system (Silberman *et al*, 1997). Second, the uPA system has a role in overcoming tissue boundaries. Recently, a physiological role for uPAR signalling in the regulation of kidney permeability has been described (Wei *et al*, 2008). Another study investigating the entry of fibrosarcoma cells into the vasculature (i.e., intravasation) points to the same direction. After inhibition of uPA by natural or synthetic inhibitors in the chorioallantoic membrane of chick embryos, both inhibitors reduced intravasation and metastasis. The authors suggest uPA activation as a key step in tumour progression (Madsen *et al*, 2006). The effects of members of the uPA system on tumour cell biology, cell migration and metastases can be reversed in *in vivo* models. Mice with a targeted deficiency for uPA or PAI-1 showed a significantly reduced tumour growth after transplantation of fibrosarcoma cells. Tumours in uPA−/− and PAI-1−/− mice displayed lower proliferative and higher apoptotic indices and displayed different neovascular morphology, as compared with WT mice (Gutierrez *et al*, 2000). In line with these results are findings in a mouse osteosarcoma model. In this *in vivo* model of tibial tumours, uPAR mRNA was expressed early (4 days), whereas uPA and PAI-1 mRNA increased as the tumour invaded the surrounding tissue (3 weeks). Interestingly, there was a preferential co-localisation of uPA, uPAR and PAI-1 mRNA to the advancing front of tibial tumours (Fisher *et al*, 2001). Furthermore, injection of an antisense uPAR inhibitor resulted in a significant reduction in tumour volumes and in total inhibition of pulmonary metastases (Dass *et al*, 2005). In another mouse model, treatment with a uPAR antibody and a recombinant pigment epithelium-derived factor that may internalise uPA/uPAR complexes led to decreased ostoeosarcoma growth and metastasis (Dass and Choong 2008).

A few studies have analysed uPA and PAI-1 antigen levels in plasma/serum and its contribution to prognosis in carcinoma patients but none have been conducted for sarcoma patients (Strojan *et al*, 1998; Miyake *et al*, 1999; Abendstein *et al*, 2000; Rha *et al*, 2000; Shariat *et al*, 2007; Iwadate *et al*, 2008; Herszényi *et al*, 2008). Strikingly, in our study, uPAR antigen levels in serum of STS patients were found to be highly and significantly associated with poor OS in Kaplan–Meier analyses and in multivariate Cox's regression analyses. This is in line with other studies that reported that high serum levels of soluble uPAR (suPAR) were significantly associated with worse survival in colorectal, prostate, ovarian and breast cancer, as well as in multiple myeloma (Sier *et al*, 1998; Brünner *et al*, 1999; Miyake *et al*, 1999; Stephens *et al*, 1999; Riisbro *et al*, 2002; Rigolin *et al*, 2003; Shariat *et al*, 2007) In contrast to cell-bound uPAR, which focuses uPA mediated plasmin formation to the cell surface, the role and source of suPAR remain to be clarified (Brünner *et al*, 1999). Holst-Hansen *et al* (1999) demonstrated that the amount of suPAR released from breast cancer cell lines was directly correlated to the number of viable cells. In addition, using a breast cancer xenograft tumour model, the authors demonstrated that the concentration of suPAR in plasma was highly correlated with tumour volume. Furthermore, Shariat *et al* (2007) found a direct association between high suPAR levels in serum and tumour burden in prostate cancer, and its decrease after tumour removal. Overall, these studies suggest that local expression/production of uPAR on tumour cells may significantly contribute to the increased levels of suPAR levels in serum of cancer patients. In our study, we observed a relatively high correlation between uPAR antigen levels in tumour tissue and serum (*r*_s_=0.54), which may indicate that suPAR levels, at least partially, derive from cancer cells. In a recent study, Riisbro *et al* (2002) did not find a correlation between uPAR levels in breast cancer tissue with that in serum of breast cancer patients, and whereas uPAR levels in serum were significantly associated with worse prognosis of breast cancer patients, uPAR levels in tumour cytosols were not. However, Meng *et al* (2006) reported that the uPAR gene status – contributing to uPAR overexpression – in breast cancer cells from blood and tumour tissue is concordant. Therefore, besides cell-bound uPAR that is shed from primary tumour tissue, other sources of suPAR should also be considered, such as blood monocytes or neutrophile granulocytes that may become activated due a systemic reaction to tumour growth/progression (Shariat *et al*, 2007), or as an association with circulating tumour cells (Mustjoki *et al*, 2000). Correlation of uPAR levels in serum with prognosis of STS patients could improve cancer detection and monitoring of cancer progression, as investigation of serum samples is more easily performed than that of cancer tissues, which is limited by tumour size, tumour heterogeneity and freezing capacities.

Most strikingly, combined high levels of uPA, uPAR and PAI-1 in tumour tissue and of uPAR in serum were found to be correlated with an additive negative effect on prognosis. However, further possibilities of affecting tumour spread and formation of metastases could be the role of the uPA system in haemostasis, as well as in inflammatory and immune processes (Mondino and Blasi, 2004). Finally, the uPA system might facilitate the recruitment of tumour cells and tumour-associated cells at the sites of metastasis using these processes.

In conclusion, co-detection of a high expression level of these uPA system members in tumour tissue and of uPAR in serum is significantly correlated with a shortened OS of STS patients, suggesting that protein expression in tumour tissue and in serum should be considered together for prognostic evaluation.

## Figures and Tables

**Table 1:** Relationship between biological marker levels in tumour tissue extracts and in serum of STS patients and clinicopathological parameters

**Patients’**		**uPA-T**	**uPAR-T**	**PAI-1-T**		**uPA-S**	**uPAR-S**	**PAI-1-S**
**characteristics**	**Cases**	**Low/high**	**Low/high**	**Low/high**	**Cases**	**Low/high**	**Low/high**	**Low/high**
No.	80				79			
*Sex* a		*P*=0.168	*P*=0.286	*P*=0.981		*P*=0.410	*P*=0.965	*P*=0.369
Male	37	22/15	21/16	18/19	35	18/17	17/18	17/18
Female	43	18/25	19/24	19/21	44	21/23	23/21	23/21
								
*Histological subtype* b		***P*<0.001**	***P*<0.001**	***P*=0.006**		*P*=0.286	***P*=0.004**	*P*=0.426
LS	21	18/3	19/2	15/6	20	13/7	15/5	12/8
MFH	17	2/15	3/14	3/14	18	7/11	4/14	9/9
FS	3	2/1	2/1	2/1	3	1/2	2/1	2/1
RMS	6	1/5	1/5	1/5	6	3/3	2/4	3/3
LMS	16	5/11	7/9	10/6	17	7/10	7/10	8/9
NS	5	3/2	1/4	3/2	4	1/3	3/1	2/2
Syn	6	5/1	4/2	4/2	6	3/3	5/1	1/5
Other	6	4/2	3/3	2/4	5	4/1	2/3	3/2
								
*Tumour grade* b		***P*<0.001**	***P*<0.001**	***P*<0.001**		*P*=0.527	***P*<0.001**	*P*=0.839
I	16	14/2	13/3	13/3	16	10/6	13/3	8/8
II	33	20/13	19/14	20/13	32	15/17	18/14	15/17
III	31	6/25	8/23	7/24	31	14/17	9/22	17/14
								
*Tumour stage* b		***P*<0.001**	***P*<0.001**	***P*=0.006**		*P*=0.390	***P*=0.019**	*P*=0.713
I	13	11/2	12/1	10/3	13	9/4	10/3	8/5
II	32	20/12	17/15	20/12	31	13/18	19/12	14/17
III	26	5/21	7/19	8/18	26	13/13	8/18	13/13
IV	9	4/5	4/5	2/7	9	4/5	3/6	5/4
								
*Complete resection* a		*P*=0.644	*P*=0.319	*P*=0.295		*P*=0.699	*P*=0.950	*P*=0.083
Radical (R0)	54	28/26	29/25	28/26	53	27/26	26/27	25/28
Not radical (R1)	26	12/14	11/15	12/14	26	12/14	14/12	15/11
								
*Localisation* b		*P*=0.230	*P*=0.133	***P*=0.038**		*P*=0.154	*P*=0.292	*P*=0.836
Extremities	52	26/26	25/27	25/27	51	23/28	25/26	26/25
Trunc wall	6	2/4	3/3	0/6	5	3/2	1/4	2/3
Head/neck	2	0/2	0/2	0/2	2	0/2	0/2	2/0
Abdomen/retro- peritoneum	20	12/18	12/8	15/5	21	13/8	14/7	10/11
*Patients follow-up* a		***P*=0.001**	***P*=0.004**	***P*=0.004**		*P*=0.957	***P*=0.013**	*P*=0.172
Alive	37	24/13	22/15	24/13	37	20/17	23/14	16/21
Dead	43	16/27	18/25	16/27	42	19/23	17/25	24/18

LS=liposarcoma; MFH=malignant fibrous histiocytoma; FS=fibrosarcoma; RMS=rhabdomyosarcoma; LMS=leiomyosarcoma; NS=neurogenic sarcoma; Syn=synovial sarcoma; T=tumour tissue extract; S=serum; uPA=urokinase plasminogen activator; STS=soft-tissue sarcoma.

a *P* for Mann–Whitney *U*-test.

b *P* for Kruskal–Wallis test.

Significant *P*-values are in bold face.

**Table 2:** Spearman's rank correlations between biological parameters

	**uPA-S**	**uPAR-S**	**PAI-1-S**	**uPA-T**	**uPAR-T**
uPAR-S	0.54^***^				
PAI-1-S	0.24^*^	0.19			
uPA-T	0.36^**^	0.49^***^	0.08		
uPAR-T	0.38^**^	0.54^***^	0.01	0.84^***^	
PAI-1-T	0.31^**^	0.46^***^	0.04	0.69^***^	0.83^***^

Spearman's rank correlation *r*_s_; ^*^P<0.05; ^**^P<0.01; ^***^ *P*<0.001.

**Table 3:** Kaplan–Meier analyses and multivariate Cox’s regression analyses: association of uPA, uPAR and PAI-1 antigen levels in tumour tissue extracts and in serum of STS patients with overall survival

**Kaplan**–**Meier analysis**				**Multivariate Cox's regression analysis**		
	***n***	**Months**	***P*-value**		**RR (95% CI)**	***P*-value**
*Tumour tissue*				*Tumour tissue*		
uPA high	40	44	**0.003**	uPA high	**2.9** (1.1–7.7)	**0.032**
uPA low	40	86				
uPAR high	40	54	**0.033**	uPAR high	2.0 (0.8–4.5)	0.108
uPAR low	40	76				
PAI-1 high	40	53	**0.004**	PAI-1 high	**2.6** (1.1–6.0)	**0.029**
PAI-1 low	40	79				
						
*Serum*				*Serum*		
uPA high	40	61	0.467	uPA high	1.2 (0.7–2.3)	0.634
uPA low	39	76				
uPAR high	40	45	**0.005**	uPAR high	**3.5** (1.5–8.3)	**0.004**
uPAR low	39	86				
PAI-1 high	40	60	0.336	PAI-1 high	1.4 (0.7–2.8)	0.333
PAI-1 low	39	71				

CI=confidence interval; PAI-1=urokinase plasminogen activator inhibitor 1; STS=soft tissue sarcoma; uPA=urokinase plasminogen activator; uPAR=urokinase plasminogen activator receptor. Significant *P*-values and RR-values are in bold face.

**Table 4:** Kaplan–Meier analyses and multivariate Cox's regression analyses for combinations of uPA, uPAR and PAI-1 antigen levels in tumour tissue and in serum of STS patients and its association with overall survival

**Kaplan–Meier analysis**	***n***	**Months**	***P*-value**	**Multivariate Cox's regression analysis**	**RR (95% CI)**	***P*** **-value**
*uPA-Tumour/uPAR-Tumour*	80			*uPA-Tumour/uPAR-Tumour*		
uPA-T low/uPAR-T low	35	75		uPA-T low/uPAR-T low		
uPA-T low/uPAR-T high	5	122		uPA-T low/uPAR-T high	0.4 (0.04–3.3)	0.364
uPA-T high/uPAR-T low	5	62		uPA-T high/uPAR-T low	1.4 (0.3–5.9)	0.688
uPA-T high/uPAR-T high	35	41	**0.007**	uPA-T high/uPAR-T high	**3.3** (1.2–9.6)	**0.026**
						
*uPA-Tumour/PAI-1-Tumour*	80			*uPA-Tumour/PAI-1-Tumour*		
uPA-T low/PAI-1-T low	31	88		uPA-T low/PAI-1-T low		
uPA-T low/PAI-1-T high	9	54		uPA-T low/PAI-1-T high	2.5 (0.7–9.7)	0.168
uPA-T high/PAI-1-T low	9	36		uPA-T high/PAI-1-T low	2.6 (0.7–9.9)	0.149
uPA-T high/PAI-1-T high	31	46	**0.004**	uPA-T high/PAI-1-T high	**3.6** (1.2–10.4)	**0.019**
						
*uPAR-Tumour/PAI-1-Tumour*	80			*uPAR-Tumour/PAI-1-Tumour*		
uPAR-T low/PAI-1-T low	29	86		uPAR-T low/PAI-1-T low		
uPAR-T low/PAI-1-T high	11	39		uPAR-T low/PAI-1-T high	**3.0** (1.0–9.2)	**0.042**
uPAR-T high/PAI-1-T low	11	44		uPAR-T high/PAI-1-T low	2.5 0.7–9.2)	0.173
uPAR-T high/PAI-1-T high	29	53	**0.014**	uPAR-T high/PAI-1-T high	**3.2** (1.1–9.4)	**0.034**
						
*uPAR-Serum/uPA-Tumour*	77			*uPAR-Serum/uPA-Tumour*		
uPAR-S low/uPA-T low	28	94		uPAR-S low/uPA-T low		
uPAR-S low/uPA-T high	11	44		uPAR-S low/uPA-T high	**4.2** (1.0–17.5)	**0.048**
uPAR-S high/uPA-T low	10	38		uPAR-S high/uPA-T low	**4.9** (1.3–18.0)	**0.016**
uPAR-S high/uPA-T high	28	44	**0.012**	uPAR-S high/uPA-T high	**5.9** (1.8–19.2)	**0.003**
						
*uPAR-Serum/uPAR-Tumour*	77			*uPAR-Serum/uPAR-Tumour*		
uPAR-S low/uPAR-T low	26	80		uPAR-S low/uPAR-T low		
uPAR-S low/uPAR-T high	12	89		uPAR-S low/uPAR-T high	0.9 (0.2–3.9)	0.929
uPAR-S high/uPAR-T low	12	59		uPAR-S high/uPAR-T low	1.9 (0.6–6.6)	0.287
uPAR-S high/uPAR-T high	27	38	**0.008**	uPAR-S high/uPAR-T high	**6.2** (1.9–20.0)	**0.002**
						
*uPAR-Serum/PAI-Tumour*	77			*uPAR-Serum/PAI-Tumour*		
uPAR-S low/PAI-T low	28	82		uPAR-S low/PAI-T low		
uPAR-S low/PAI-T high	10	81		uPAR-S low/PAI-T high	1.9 (0.5–6.4)	0.327
uPAR-S high/PAI-T low	11	49		uPAR-S high/PAI-T low	2.6 (0.8–8.9)	0.115
uPAR-S high/PAI-T high	28	39	**0.007**	uPAR-S high/PAI-T high	**5.8** (1.9–17.0)	**0.001**

CI=confidence interval; PAI-1=urokinase plasminogen activator inhibitor 1; STS=soft-tissue sarcoma; uPA=urokinase plasminogen activator; uPAR=urokinase plasminogen activator receptor. Significant *P*-values and RR-values are in bold face.
